# Retraction Note: Fractional boundary element solution of three-temperature thermoelectric problems

**DOI:** 10.1038/s41598-024-63283-6

**Published:** 2024-05-29

**Authors:** Mohamed Abdelsabour Fahmy, Mohammed M. Almehmadi, Fahad M. Al Subhi, Ayesha Sohail

**Affiliations:** 1 Department of Mathematics, Jamoum University College, Umm Al-Qura University, Alshohdaa, Jamoum, Makkah, 25371 Saudi Arabia; 2 Faculty of Computers and Informatics, Suez Canal University, New Campus, Ismailia, 41522 Egypt; 3 Department of Mathematical Sciences, Faculty of Applied Sciences, Umm Al-Qura University, Makkah, 24381 Saudi Arabia; 4 Department of Mathematics, Comsats University Islamabad, Lahore Campus, Lahore, 54000 Pakistan

Retraction of: *Scientific Reports* 10.1038/s41598-022-10639-5, published online 26 April 2022

The Editors have retracted this Article.

After publication concerns were raised about the validation methodology used and the inclusion of irrelevant citations and a high number of self-citations. The Editors have not received a satisfactory response from the Authors to these concerns nor have the Authors provided the underlying data and code when requested.

The Editors therefore no longer have confidence in the results and conclusions presented.

None of the Authors has responded to the correspondence from Editors about this retraction.

